# Genome-Wide Analysis of Tomato *SlCCD* Genes and the Role of *SlCCD11* in Enhancing Salt Tolerance

**DOI:** 10.3390/plants15020300

**Published:** 2026-01-19

**Authors:** Caiting An, Zesheng Liu, Mengkun Liu, Qianbin Li, Qi Wang, Min Cao, Xinmeng Geng, Chunlei Wang

**Affiliations:** College of Horticulture, Gansu Agricultural University, Lanzhou 730070, China; 15394090106@163.com (C.A.); lzs0724@163.com (Z.L.); 15763006751@163.com (M.L.); 13609382674@163.com (Q.L.); 15693303950@163.com (Q.W.); 17865688829@163.com (M.C.); 15933695972@163.com (X.G.)

**Keywords:** tomato seedling, the *SlCCD* gene family, virus-induced gene silencing, carotenoids degradation, abscisic acid, salt stress

## Abstract

Tomato (*Solanum lycopersicum* L.) is an important horticultural crop. Carotenoid cyclase dioxygenase (CCD) is an enzyme responsible for cleaving carotenoids, which is involved in regulating plant growth and response to abiotic stresses. However, the role of *SlCCDs* in tomato stress resistance remains unclear. This study used the tomato variety ‘Micro-Tom’ as the material to investigate the function of *SlCCDs* in stress responses. Through whole-genome analysis, a total of 12 *SlCCDs* members (*SlCCD1*–*SlCCD12*) were identified. Systematic evolutionary analysis classified them into four branches, and members within the same branch maintained a conserved structure. The promoter analysis revealed that *SlCCDs* contain multiple hormones and stress response elements. The qRT-PCR analysis revealed that *SlCCD11* was the most highly expressed gene in the leaves. In addition, multiple *SlCCDs* showed significant responses to abscisic acid (ABA), methyl jasmonate (MeJA), light, and sodium chloride (NaCl) treatments. Among them, the expression of *SlCCD11* significantly increased under salt stress. By silencing *SlCCD11* using virus-induced gene silencing (VIGS) technology, it was found that the chlorophyll content, antioxidant enzyme activity, and ABA-related gene expression in the TRV:*SlCCD11* plants under salt stress were all lower than the control samples, while the carotenoid content and ROS accumulation were higher. This indicates that *SlCCD11* is a positive regulatory factor for salt stress. In conclusion, this study systematically analyzed the *SlCCD* gene family and revealed the positive role of *SlCCD11* in tomato response to salt stress, providing a candidate gene for genetic improvement of crop stress resistance.

## 1. Introduction

Tomato (*Solanum lycopersicum* L.) is an important horticultural crop and a model plant in the *Solanaceae* family, and salt stress is one of the major environmental stresses affecting its cultivation [1]. When plants were subjected to salt stress, the intracellular ion concentration and osmotic pressure increased, which inhibited normal growth, development, and life processes, and could even lead to wilting and death [2]. When plants are exposed to salt stress, they will evolve complex regulatory mechanisms to cope with it. For example, abscisic acid (ABA) functions by activating stress responses and restricting plant growth to counter osmotic stress [3]. However, the biosynthesis of ABA originates from the cleavage of carotenoids. Carotenoids are important lipid-soluble compounds comprising a large family of over 700 diverse structures [4,5]. In plants, carotenoids serve as a core substance for absorbing light energy and conducting photosynthesis. They work synergistically with chlorophyll to mitigate photodamage and oxidative stress under strong light conditions [6].

Carotenoid cleavage oxygenases (CCOs) are a family of enzymes that catalyze the oxidative cleavage of carotenoids to produce apocarotenoids and play a central role in plant carotenoid metabolism [7]. The endogenous level of carotenoids is regulated by the dynamic balance of their synthesis and degradation pathways. The degradation process involves both enzymatic and non-enzymatic pathways [8]. Enzymatic cleavage was the key pathway for the production of apocarotenoids [9]. This process was primarily mediated by the carotenoid cleavage dioxygenase (CCD) family, which acted through the oxidative cleavage of carotenoids at their conjugated double bonds to generate apocarotenoid products with diverse biological functions [10], such as volatile scent compounds (α-ionone, β-ionone), pigments (bixin, crocin), and plant hormones like ABA, and strigolactones (SLs) [11]. At present, nearly 100 types of apocarotenoids have been identified in tobacco (*Nicotiana tabacum* L.). During the aging and drying processes of the leaves, the carotenoid content decreases due to decomposition [6]. Based on the presence of an epoxy group and a 9-cis double bond in their substrates, CCOs enzymes were categorized into two subfamilies, 9-cis-epoxycarotenoid dioxygenases (NCEDs) and *CCDs* [12]. For example, in *Arabidopsis thaliana*, nine *CCO* gene family members were identified, including four carotenoid cleavage dioxygenases (*AtCCD1*, *AtCCD4*, *AtCCD7*, and *AtCCD8*) and five *NCED* (*AtNCED2*, *AtNCED3*, *AtNCED5*, *AtNCED6*, and *AtNCED9*) [7]. Among them, the reaction catalyzed by NCED was the rate-limiting step in the biosynthesis of ABA. This further affects the plant’s tolerance to abiotic stress such as drought and salt stress [13]. Therefore, many studies have focused on the regulatory mechanisms of NCED when it responds to stress conditions. However, CCD enzymes do not possess a unique substrate cleavage site. Different CCDs are able to cleave or recognize specific carotenoid or apocarotenoid substrates [14]. And plants possess several types of CCD enzymes, including CCD1, CCD2, CCD4, CCD7, CCD8, and CCD10 [5,14]. For example, *AtCCD1* and *AtCCD4* primarily cleave the 9,10 double bonds of carotenoids. They catalyzed the conversion of C_40_ carotenoids and C_27_ iridoids into small volatile molecules such as C_13_ β-carotenc and C_14_ geranyl acetone, indicating that these two enzymes played a key role in the formation of plant aroma [15]. In *Crocus sativus* L. (*Iridaceae*), *CsCCD1*, together with *CsCCD2* and *CsCCD4*, is involved in carotenoid metabolism, a process essential for producing the apocarotenoids that give the spice’s unique color, taste, and aroma [16].

The various subgroups within the *CCD* family performed diverse functions, which were crucial for plant growth and development, their responses to stresses, and the formation of specific carotenoid-derived products [4]. For example, in *Dendrobium officinale*, the *DoCCD1* gene was able to directly produce β-ionone, which emits a violet aroma, by cleaving β-carotene; thus, identifying CCD1 as the functional enzyme for synthesizing this key aromatic compound [17]. Similarly, during the tea (*Camellia sinensis*) withering process, the content of β-ionone increased, further underscoring the central role of its CCD4 enzyme in producing this flavor compound [18]. *CCD* members are also widely implicated in pigment formation, influencing the coloration of flowers, flesh, and fruit peel. For instance, in citrus (*Citrus clementina*), CCD4b1 utilizes β-cryptoxanthin and zeaxanthin as substrates, cleaving them at the 7, 8 double bonds to generate unique C_30_ apocarotenoids that enhance peel coloration [19]. CCD7 and CCD8 were mainly involved in the biosynthesis of SLs in plant vegetative organs such as roots and stems. As important plant signaling molecules, SLs not only regulated lateral shoot growth, root development, and interactions with soil microorganisms but also play a role in regulating plant growth and development [20]. Furthermore, the *CCD* family is also extensively involved in plant response to abiotic stress. In soybeans (*Glycine max*), the expression levels of the *CCD* genes significantly changed under salt, drought, low-temperature, and high-temperature stress conditions, indicating that they directly participate in the abiotic stress adaptation process of soybeans [21]. Similarly, the expression levels of *CCD1* and *CCD4* in rapeseed (*Brassica napus* L.) [22], and *MdCCD* members in apple (*Malus domestica*) [23] also significantly adjusted under drought or salt stress conditions, further confirming the universal role of *CCDs* in plants’ abiotic stress responses.

In recent years, with the completion of plant genome sequencing, genes belonging to the *CCD* family have been identified across various plant species [24]. However, there is a lack of systematic identification of CCD members in tomatoes. In addition, although *CCDs* have significant resistance to abiotic stress, including salt stress, their mechanism for dealing with salt stress in tomato is still not well understood [4]. In this study, we systematically identified the tomato *SlCCDs* gene family. Through qRT-PCR expression analysis, we revealed its regulatory role in abiotic stress responses. Furthermore, the molecular mechanism by which the *SlCCD11* gene in tomato participates in the response to salt stress was further investigated. The results also offered valuable insights into its evolutionary patterns and established a theoretical basis for further exploration of the functional roles of *SlCCDs* in tomato.

## 2. Results

### 2.1. Identification of SlCCD Family Members in Tomato

In our study, twelve *SlCCD* genes were identified in tomato through homology comparison and subsequently named *SlCCD1* to *SlCCD12* based on their chromosomal localization. The tomato *SlCCD* protein family exhibits a broad spectrum of molecular weights, ranging from 9548.05 Da (*SlCCD3*) to 76,853.95 Da (*SlCCD4*) and amino acid lengths, varying from 86 to 678 residues. Physicochemical property predictions categorize *SlCCD4*, *SlCCD5*, *SlCCD10*, and *SlCCD11* as basic proteins (pI > 7), while *SlCCD1*, *SlCCD2*, *SlCCD3*, *SlCCD6*, *SlCCD7*, *SlCCD8*, *SlCCD9*, and *SlCCD12* are classified as acidic proteins (pI < 7). Moreover, hydrophobicity assessments reveal that all members of the tomato *SlCCDs* family are hydrophilic proteins, with *SlCCD10* exhibiting the most negative hydrophobicity value of −0.155 (Table 1).

### 2.2. Conserved Motifs and Chromosomal Localization Analysis of SlCCD Family Members

**Figure 1:**
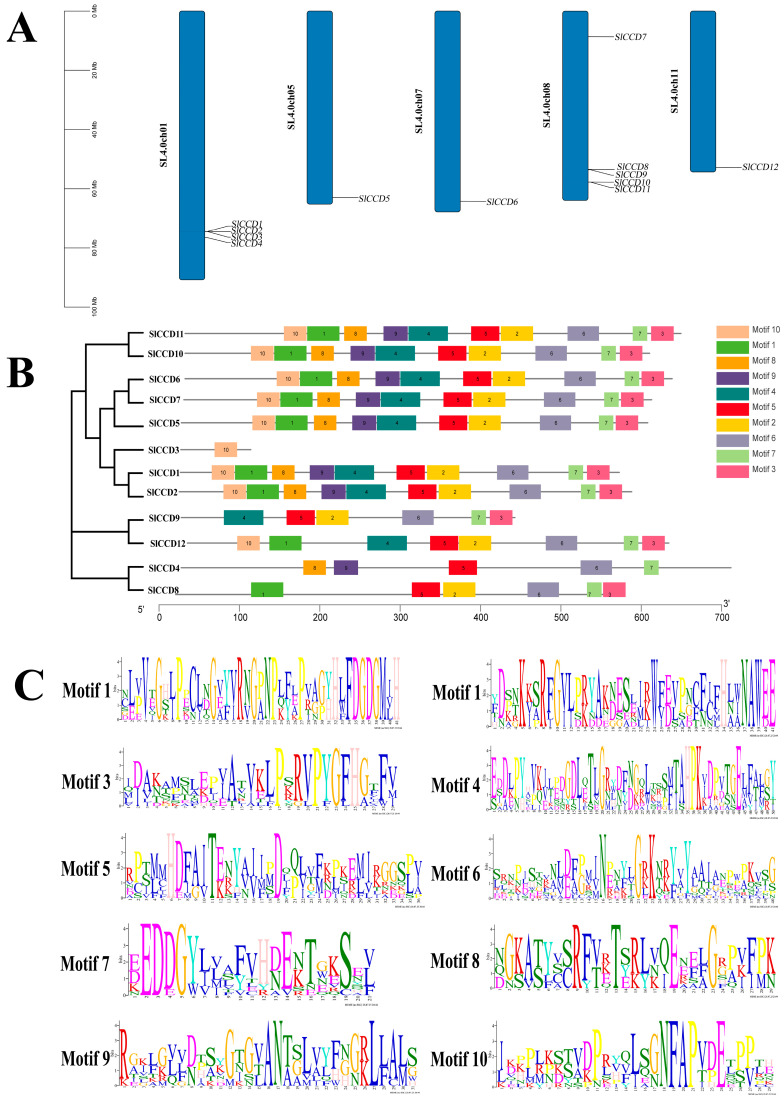
Analysis of the characteristics of the *SlCCD* gene family members. (**A**) Chromosomal localization of tomato *SlCCD* family genes. (**B**) Conserved motifs of tomato *SlCCD* proteins. (**C**) Motifs 1–10 are shown, with each conserved motif represented as a sequence logo where the amino acid sequence is depicted by stacked letters at each position.

To investigate the evolutionary characteristics of the SlCCD gene family in tomato, we analyzed the chromosomal locations and conserved motif features of its members and visualized the results with TBtools (v2.034) software. As illustrated in Figure 1A, *SlCCDs* are unevenly distributed across chromosomes. Specifically, *SlCCD1*, *SlCCD2*, *SlCCD3*, and *SlCCD4* are located on chromosome 1; *SlCCD5*, *SlCCD6*, and *SlCCD12* are found on chromosomes 5, 7, and 11, respectively; and *SlCCD7*, *SlCCD8*, *SlCCD9*, *SlCCD10*, and *SlCCD11* are situated on chromosome 8. Additionally, we identified 10 conserved motifs within tomato CCD proteins. Notably, only Motif 10 is absent in SlCCD3. Furthermore, Motifs 1, 2, 3, 4, and 10 are missing in SlCCD4. SlCCD8 lacks Motifs 4, 8, 9, and 10, while *SlCCD9* is missing Motifs 1, 8, 9, and 10. *SlCCD12* does not contain Motifs 6, 8, and 9. However, all other members retain these 10 conserved motifs (Figure 1B). Moreover, the amino acid sequences of the different conserved motifs are represented by a series of letters at each position (Figure 1C).

### 2.3. SlCCD System Evolutionary Tree Analysis

**Figure 2:**
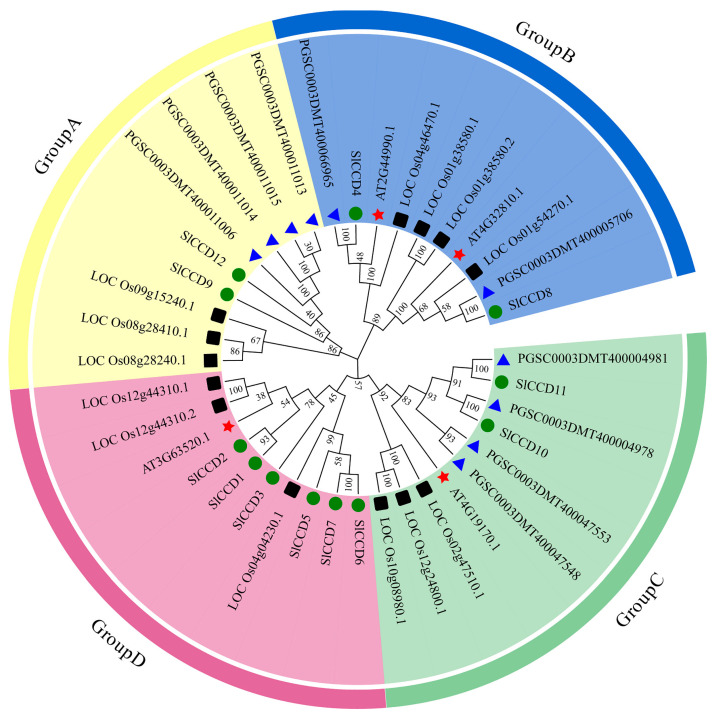
An unrooted phylogenetic tree for the CCD gene family was constructed using the maximum likelihood method, comprising sequences from 12 tomato (Sl), 4 *A. thaliana* (At), 8 potatoes (St), and 10 rice (Os) protein sequences. The four subgroups are color-coded, with each assigned a unique shape to represent their respective species. The black square represents rice, the blue triangle represents potato, the red star represents *A. thaliana*, and the green circle represents tomatoes. The numbers on the nodes in the phylogenetic tree indicate the percentage of branch confidence based on resampling validation. The numbers on the nodes in the phylogenetic tree indicate the percentage of branch confidence based on resampling validation.

To gain further insights into the evolutionary dynamics of the *SlCCD* family in tomato, we conducted a comparative analysis of 39 CCD protein sequences from tomatoes, potatoes, rice, and *A. thaliana*, followed by the construction of phylogenetic trees (Figure 2). The CCD proteins across these plant species were categorized into four distinct subgroups based on their sequence homology. Group A comprises nine members, notably *SlCCD9* and *SlCCD12*. Group B encompasses 10 members, including *SlCCD4* and *SlCCD8*. Group C is represented by 10 members, including *SlCCD10* and *SlCCD11*. Group D, with 10 members, includes *SlCCD1*, *SlCCD2*, *SlCCD3*, *SlCCD5*, *SlCCD6*, and *SlCCD7*. Furthermore, within Group D, *SlCCD1* and *SlCCD2* cluster together, as do *SlCCD6* and *SlCCD7*, indicating a close relationship between branches. Notably, the phylogenetic analysis reveals a close evolutionary relationship between *SlCCDs* and *StCCDs*.

### 2.4. SlCCD Cis-Acting Element Analysis

**Figure 3:**
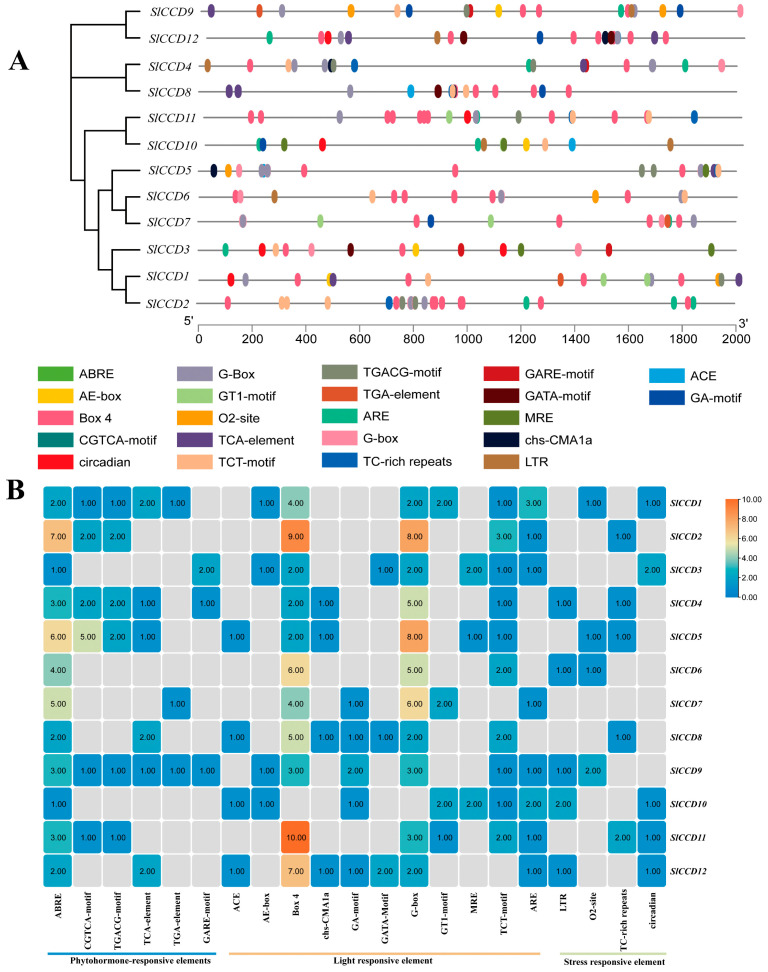
Analysis of *cis*-acting elements in tomato *SlCCD* genes. (**A**) Schematic representation of *cis*-elements composition within *SlCCD* gene promoters. Different colors denote distinct types of *cis*-elements; the length and position of each gene are drawn to scale. (**B**) Quantitative distribution of *cis*-element types across the *SlCCD* gene family. Color intensity and numerical values indicate the abundance of each element type.

We analyzed the promoter sequences of 12 *SICCD* genes (from −2000 bp to −1 bp) to identify *cis*-acting elements. A total of 22 major *cis*-acting elements were found in the promoter regions of tomato *SICCD* family members. Based on their characteristics, these elements can be categorized into three groups: light response elements, hormone response elements, and stress response elements. As illustrated in Figure 3A, the ABRE component exhibited extensive responsiveness across the entire family. In contrast, TCA elements demonstrated minimal responsiveness to hormones. Box4 and G-box elements were present in all tomato *SICCD* genes except for *SICCD10*, with the highest concentration observed in *SICCD11*. Notably, *SlCCD2* exhibited the greatest abundance of Box4 and G-box elements, totaling 17 (Figure 3B). Additionally, ACE, AE-box, and chs-CMA1a were identified as the three elements least responsive to light. Among stress response elements, ARE was the most prominent, with the exception of *SICCD4*, *SICCD5*, *SICCD6*, and *SICCD8* (Figure 3B).

### 2.5. Tissue-Specific Expression Analysis of SlCCDs

**Figure 4:**
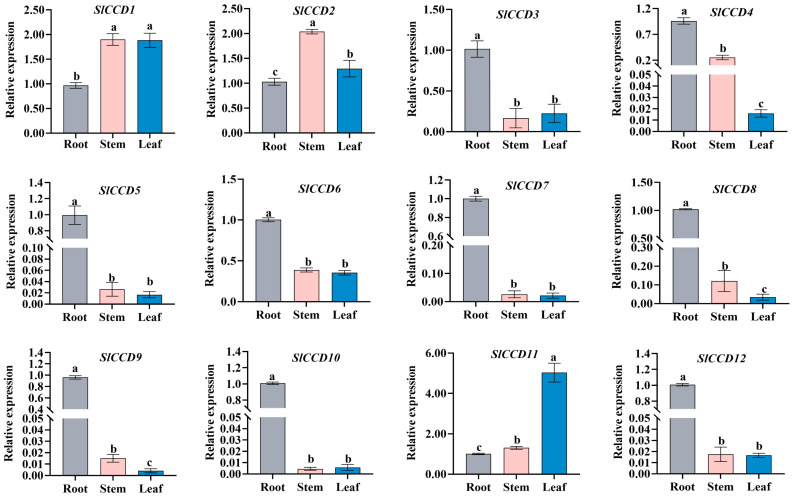
Analysis of expression levels of the tomato *SlCCD* gene family members across different tissues during growth and development. The expression levels of each gene are presented as mean ± standard error (*n* = 3), and the error bars represent the standard error (SE) of three biological replicates. Duncan’s multiple range test was used for statistical analysis (*p* < 0.05), and different lowercase letters indicate statistically significant differences among groups.

To examine the tissue-specific expression patterns of the *SlCCD* gene family in tomato, we initially conducted an in-system analysis of 14 tomato tissues utilizing an online database (Figure S3). Given that the database included only 11 family members, we subsequently assessed the expression levels of 12 *SlCCD* genes across various tissues of tomato seedlings through qRT-PCR, thereby providing a comprehensive evaluation of the expression profile of this gene family (Figure 4). The qRT-PCR findings indicated that *SlCCD3*, 4, 5, 6, 7, 8, 9, 10, and 12 exhibited the highest expression levels in roots, while *SlCCD2* expression peaked in stems, and *SlCCD11* was predominantly expressed in leaves.

### 2.6. Expression Analysis of SlCCD Genes in Tomato Under Hormonal and Abiotic Stresses

**Figure 5:**
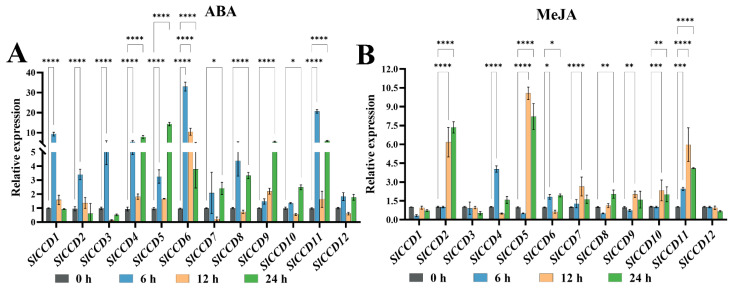
Expression of *SlCCD* genes in response to ABA and MeJA treatments. (**A**) Expression patterns under ABA treatments. (**B**) Expression patterns under MeJA treatments. Asterisks (*) indicate that the expression level of the stress group is significantly different from that of the control group (*** *p* < 0.001, ** *p* < 0.01, * *p* < 0.05, and **** *p* < 0.0001, one-way ANOVA, and Tukey’s test). Samples treated for 0 h were used as the control samples.

To investigate the response patterns of *SlCCD* genes to various plant hormones and abiotic stresses, we initially assessed the expression levels of 12 *SlCCD* genes in tomato plants subjected to ABA and MeJA treatments. ABA contrast significantly suppressed the expression of *SlCCD7*, *SlCCD10*, and *SlCCD12*. Conversely, ABA treatment for 0–6 h significantly elevated the expression levels of *SlCCD1*, *SlCCD2*, *SlCCD3*, *SlCCD4*, *SlCCD5*, *SlCCD6*, *SlCCD8*, and *SlCCD11* (Figure 5A). Under MeJA treatment, no significant differences were observed in the expression levels of *SlCCD1*, *SlCCD3*, and *SlCCD12* (Figure 5B). Furthermore, the expression levels of *SlCCD2*, *SlCCD5*, *SlCCD7*, *SlCCD9*, *SlCCD10*, and *SlCCD11* significantly increased after 12 h of MeJA treatment.

**Figure 6:**
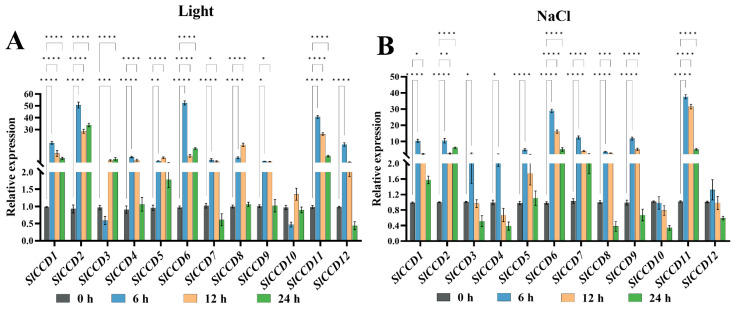
Expression of *SlCCD* genes in response to Light and NaCl treatments. (**A**) Expression patterns under light treatments. (**B**) Expression patterns under NaCl treatment. Asterisks (*) indicate that the expression level of the stress group is significantly different from that of the control group (*** *p* < 0.001, ** *p* < 0.01, * *p* < 0.05, and **** *p* < 0.0001, one-way ANOVA, and Tukey’s test). Samples treated for 0 h were used as the control samples.

In addition, we analyzed the *SlCCD* genes under light and NaCl treatments. However, there was no significant difference between *SlCCD9* and *SlCCD10* under light treatment (Figure 6A). Under NaCl treatment, the expression patterns of *SlCCD* genes reveal distinct trends (Figure 6B). Most *SlCCD* genes, including *SlCCD1*, *SlCCD2*, *SlCCD5*, *SlCCD6*, *SlCCD7*, *SlCCD8*, and *SlCCD11*, exhibit time-dependent fluctuations over the 0–24 h period. For example, their expression levels typically increase initially, as seen with *SlCCD6* at 6 h, before declining or fluctuating in subsequent intervals. Notably, *SlCCD11*, which is characterized by the most significant expression during the 0–24 h treatment, demonstrates the most pronounced variation: its expression peaks sharply at 6 h, significantly exceeding levels observed at other time points, and remains relatively elevated at 12 h and 24 h compared to the initial 0 h. In contrast, *SlCCD3*, *SlCCD4*, *SlCCD10*, and *SlCCD12* exhibit minimal changes across all time points, showing no significant differences in expression throughout the NaCl treatment.

### 2.7. Effect of Transient Silencing of SlCCD11 on the Growth Phenotype of Tomato Seedlings Under Salt Stress

**Figure 7:**
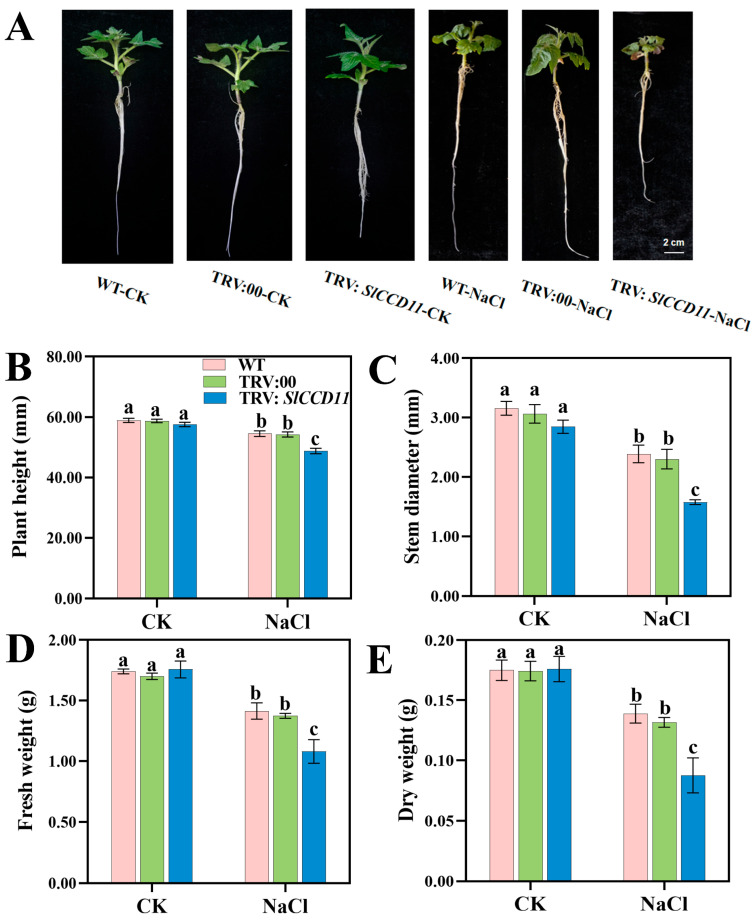
Effects of instantaneous silencing of *SlCCD11* on seedling growth under salt stress. (**A**) Photos of plants treated with 150 mM NaCl for 7 d. (**B**) plant height, (**C**) Stem diameter, (**D**) Fresh weight, (**E**) Dry weight. The data in the figure are all the average ± SD of three repeated experiments, indicating the significance of differences is represented by letters (*p* < 0.05).

**Figure 8:**
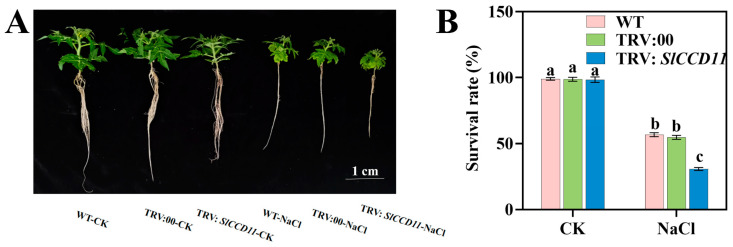
Effect of transient silencing of *SlCCD11* on the survival rate of tomato plants under salt stress. (**A**) Photos of plants treated with 150 mM NaCl for 14 d. (**B**) Survival rate. The data in the figure are all the average ± SD of three repeated experiments, indicating the significance of differences is represented by letters (*p* < 0.05).

To investigate the impact of *SlCCD11* on tomato growth and development under salt stress, we constructed a transient silencing vector for silenced seedlings (TRV: *SlCCD11*) and successfully achieved transient silencing of *SlCCD11* in the tomato variety ‘Micro Tom’ using *Agrobacterium*-mediated infiltration technology. We compared plant height, stem diameter, fresh weight, dry weight, leaf area, and total root length among the wild type (WT), TRV:00, and *SlCCD11* silenced seedlings (TRV: *SlCCD11*). As illustrated in Figure 7, under non-salt treatment conditions (CK), there were no significant differences in the phenotypes of the WT, TRV:00, and TRV:SlCCD11 plants (Figure 7A–C). However, after the application of salt stress, TRV:SlCCD11 plants displayed more pronounced chlorosis and reduced growth (Figure 7A). Specifically, plant height (Figure 7B), stem diameter (Figure 7C), fresh weight (Figure 7D), and dry weight (Figure 7E) in TRV:SlCCD11 plants decreased by 10.57%, 34.03%, 23.57%, and 36.96%, respectively, compared with TRV:00. Furthermore, we found that the survival rate of TRV:SlCCD11 plants 14 d of salt treatment was 54.1% lower than that of TRV:00 (Figure 8).

### 2.8. SlCCD11 Modulates Chlorophyll and Carotenoid Accumulations Under Salt Stress

**Figure 9:**
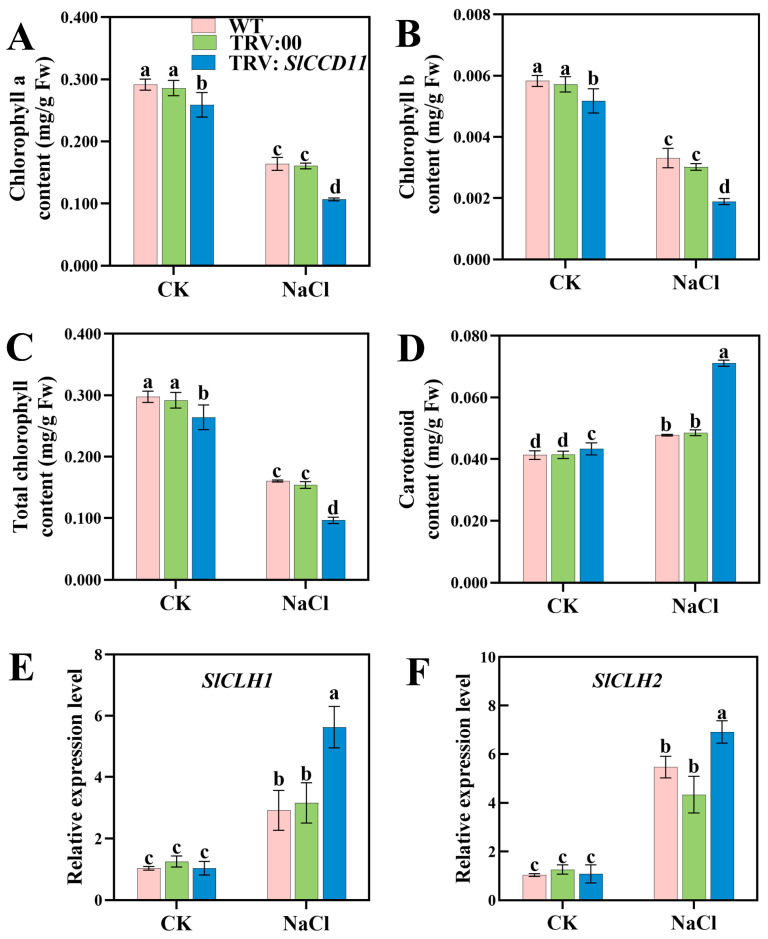
Effects of instantaneous silencing of *SlCCD11* on chlorophylls and carotenoid accumulations. (**A**) Chlorophyll a content; (**B**) Chlorophyll b content; (**C**) Total Chlorophyll content. (**D**) Carotenoid content (**E**), *SlCLH1* relative expression level (**F**), and *SlCLH2* relative expression level. The data in the figure are all the average ± SD of three repeated experiments, indicating the significance of differences is represented by letters (*p* < 0.05).

To further investigate the impact of *SlCCD11* on photosynthetic pigment accumulation in tomato seedlings subjected to salt stress, we measured chlorophyll and carotenoid contents in this study. As illustrated in Figure 9, under CK, the chlorophyll a, chlorophyll b, and total chlorophyll contents in TRV:*SlCCD11* plants did not differ significantly from those in WT and TRV:00 plants. However, following NaCl treatment, TRV:*SlCCD11* plants exhibited a significant reduction in chlorophyll a and chlorophyll b compared to TRV:00 plants, with decreases of 34.85% and 42.88%, respectively (Figure 9A–F). In contrast, carotenoid content increased significantly by 32.8% in TRV:*SlCCD11* plants relative to TRV:00 plants (Figure 9C). Furthermore, under CK conditions, we observed no significant differences in the expression of chlorophyll degradation genes, including Chlorophyllase, CLH1 (*SlCLH1*), and *SlCLH2*, among WT, TRV:00, and TRV:*SlCCD11* plants. Under salt stress, the expression of these genes in TRV:*SlCCD11* plants was significantly altered compared to TRV:00 plants, with increases of 43.9% and 37.3%, respectively (Figure 9E,F).

### 2.9. SlCCD11 Promotes SlNCED Expression Under Salt Stress

**Figure 10:**
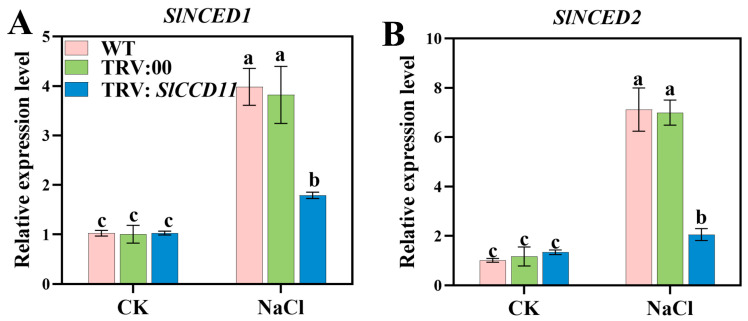
Effects of transient *SlCCD11* silencing on ABA synthesis-related gene expression under salt stress. (**A**) *SlNCED1* relative expression level. (**B**) *SlNCED2* relative expression level. The data in the figure are all the average ± SD of three repeated experiments, indicating the significance of differences is represented by letters (*p* < 0.05).

We further analyzed the relative expression levels of *SlNCED1* and *SlNCED2*, two key genes involved in ABA biosynthesis. As shown in Figure 10, in CK, no significant differences were observed in the expression of *SlNCED1* and *SlNCED2* among the WT, TRV:00, and TRV:SlCCD11 plants. Under salt stress, the expression levels of *SlNCED1* and *SlNCED2* in TRV:SlCCD11 plants were reduced by 55.05% and 71.17%, respectively, compared to those in TRV:00 plants (Figure 10A,B).

### 2.10. Silencing of SlCCD11 Aggravates Oxidative Damage Under Salt Stress

**Figure 11:**
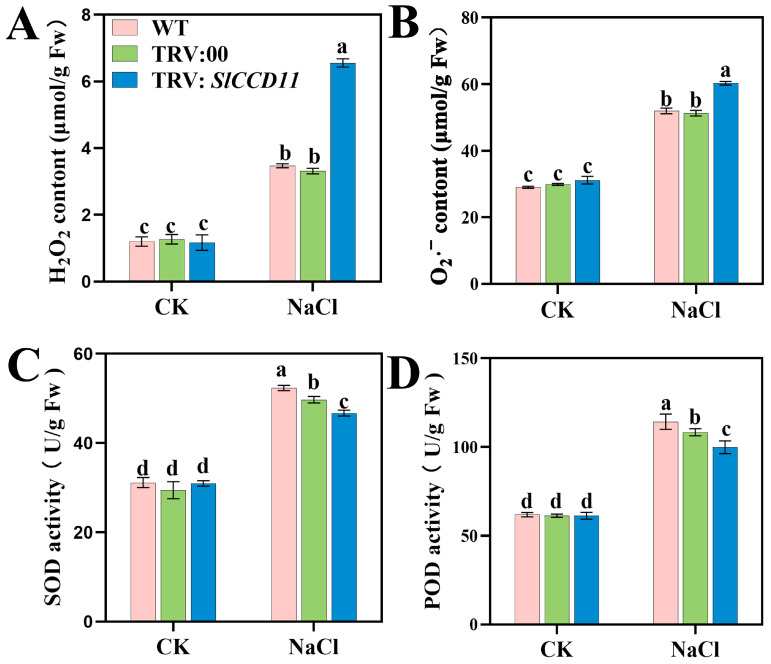
Effect of transient silencing of *SlCCD11* on ROS accumulation in tomato. (**A**) H_2_O_2_ contents, (**B**) O_2_·^-^ contents, (**C**) SOD activity, (**D**) POD activity. The data in the figure are all the average ± SD of three repeated experiments, indicating the significance of differences is represented by letters (*p* < 0.05).

Salt stress may induce oxidative stress responses in plants, leading to increased oxidative damage. As illustrated in Figure 11, under CK conditions, the levels of H_2_O_2_, O_2_·^−^, SOD, and POD in TRV:SlCCD11 plants did not differ significantly from those in WT and TRV:00 plants. However, following NaCl treatment, there was a significant increase in the accumulation of H_2_O_2_ and superoxide anion in tomato seedlings. Compared to TRV:00 plants, TRV:SlCCD11 plants exhibited markedly higher levels of oxidative products. Specifically, the H_2_O_2_ content increased by 47.05%, while the O_2_·^−^ content rose by 13.77% (Figure 11A,B). Additionally, as shown in Figure 11, the activities of SOD and POD in TRV:SlCCD11 plants subjected to NaCl treatment were lower than those in TRV:00 plants, with reductions of 10.7% and 12.5%, respectively (Figure 11C,D).

## 3. Discussion

CCD is a small gene family that catalyzes the hydrolysis of carotenoids to produce aromatic compounds and plant hormones, thereby influencing the aroma, color, and various physiological processes of plants [25]. CCDs are widely distributed in plants and play crucial roles in regulating plant growth, development, and responses to abiotic stresses [4]. Furthermore, the number of CCD genes varies significantly among different species. Specifically, 10 members were identified in ornamental woody *Prunus mume* [26], 10 in peach (*Prunus persica* L. Batsch) [27], and 21 in apple [23]. In herbaceous plants, there are 30 *CCD* members in rapeseed [19] and 19 in tobacco [28]. Additionally, in the *Cucurbitaceae* plants, there are 10, 9, 9, 13, 8, and 8 genes, respectively, in watermelon (*Citrullus lanatus*), melon (*Cucumis melo*), cucumber (*C. sativus*), pumpkin (*Cucurbita moschata*), gourd (*Lagenaria siceraria*), and wax gourd (*Benincasa hispida*) [29]. In this study, genome-wide analysis identified a total of 12 *SlCCD* members in tomato, a member of the *Solanaceae* family (Table 1). This number is less than that in tobacco and rapeseed, but is similar to the number in watermelon and cucumber. This seems to be related to the size of the genome and the number of whole-genome duplications [30]. For example, one study identified 33, 31, 16, and 15 *CCD* genes, respectively, in the allotetraploid cottons *G. hirsutum* and *G. barbadense*, as well as their two diploid relatives [31]. In addition, chromosomal localization analysis revealed that *SlCCD* gene family members are unevenly distributed across the genome and exhibit significant clustering (Figure 1A). Similarly, in *Cucurbita maxima*, 15 *CcCCO* genes were discovered, including 11 *NCED* and 4 *CCD* genes, which are scattered across different chromosomes [16]. This distribution pattern may reflect distinct evolutionary histories and functional divergence. Furthermore, it was found that the conserved motifs in *SlCCD1*, *SlCCD2*, *SlCCD5*, *SlCCD6*, *SlCCD7*, *SlCCD10*, and *SlCCD11* were identical (Figure 1B,C). Therefore, the size, distribution, and structural conservation of the *SlCCD* family genes can be attributed primarily to differences in genome size and the occurrence of whole-genome duplication events among species.

The system evolutionary tree can cluster based on the sequence similarity of homologous members, and in combination with their functional or structural characteristics, it can further assist in determining the different subfamilies within the family. As Zhou et al. (2019) reported in tobacco, different subfamilies exhibit significant differences in gene patterns and structures, which suggests the functional differentiation might exist among the AtCCD subgroups [28]. Based on the crucial role of phylogenetic analysis in elucidating gene functions and origins, we constructed a phylogenetic tree that included *CCD* proteins from tomatoes, *A. thaliana*, rice, and potatoes [32]. The results demonstrated that all the members were divided into four main groups (Figure 2), among which the tomato *SlCCD* members were most abundant in Group D. It is worth noting that *SlCCD1* and *SlCCD2*, as well as *SlCCD6* and *SlCCD7*, are, respectively, clustered in the same branch, suggesting that they may have originated from a recent gene duplication event and possess potential for functional redundancy or coordinated regulation [33]. In addition, the 12 *SlCCD* members were unevenly distributed among these four groups. This suggests a close evolutionary relationship between *SlCCDs* and the other three plant *CCD* members. Particularly importantly, *SlCCDs* and *StCCDs* were most closely related phylogenetically. Based on the principle that high conservation of gene structure typically predicts functional consistency, this result implies that *SlCCD* and *StCCD* members may share similar biological functions. However, further verification concerning their conserved protein functions is still required.

*Cis*-acting elements, as important regulators of gene expression, achieve regulation of the related genes by offering binding sites to the specific transcription factors [34]. Therefore, the present study analyzed the *cis*-acting elements in the promoter regions of tomato *SlCCD* genes. The results showed that ABRE, light-responsive, and abiotic stress-responsive elements are present in the promoters of tomato *SlCCD* genes (Figure 3A), which is consistent with the results of Wei et al. (2016) [13]. Thus, the *SlCCD* gene family may have a broad and important responsiveness to these light, plant hormones, and abiotic stress responses in tomato. Furthermore, qRT-PCR analysis showed that both ABA and light treatments significantly upregulated the expression of most *SlCCD* genes (Figure 5 and Figure 6A). In particular, NaCl treatment had the most pronounced inductive effect on the expression levels of the majority of the *SlCCD* family members (Figure 6B). These expression profiles were highly consistent with the aforementioned analysis of *cis*-acting elements in the *SlCCD* promoters, providing direct evidence for the potential role of these predicted elements in regulating *SlCCD* gene expression.

Through tissue-specific expression analysis, it was found that *SlCCD2* and *SlCCD6* have higher expression levels during fruit ripening (Figure S1). Furthermore, studies have shown that the *CCD* family is also expressed in other tissues. [23]. For instance, *CmCCD4* in melon was highly expressed in stems and leaves, while *MdCCD4* in apple was highly expressed in leaves. Similarly, in our study, *SlCCD1* displayed a distinct expression pattern in the stems and leaves of tomato seedlings. It is worth noting that *SlCCD11* is specifically expressed in leaves, whereas its expression levels in the root and stem were relatively low. This indicates that plant *CCDs* may play a significant role in the growth of leaves (Figure 4). Furthermore, *SlCCD7* is accumulated in the roots, which is consistent with the results of high expression of *CmCCD7* in cucumbers and *NtCCD7* in tobacco in the roots. Hence, the *CCD* family genes may be widely present in most kinds of plant roots. The expression of these genes in root tissues may play a role in the physiological processes related to the root system. However, their true functions still need to be further confirmed through subsequent experiments to verify the gene functions.

Salt stress inhibited plant growth through various pathways such as osmotic stress, ionic toxicity, and oxidative stress [1]. Research indicates that saline irrigation enhances the biosynthesis of 2-acetyl-1-pyrroline (2-AP) in fragrant rice by upregulating the proline metabolism pathway and, when combined with a non-functional *BADH2* gene, further promotes aroma accumulation by alleviating synthesis inhibition. However, while saline irrigation enhances aroma, it also leads to reductions in brown rice rate, milled rice rate, head rice rate, and grain amylose content. Ultimately, yield loss and comprehensive deterioration of grain quality emerge as the primary negative consequences of salt stress [35]. Furthermore, under salt stress, salt ions taken up by the plant root system are transported with nutrients from the plant body to the above-ground parts of the plant, such as stems and leaves, resulting in stunted plant growth. For example, in wheat (*Triticum aestivum* L.), elevated salt content significantly reduced stem height, decreased main stem diameter, impaired the differentiation of young spikelets, and consequently lowered crop yield [36]. The leaves of apple seedlings subjected to salt stress appeared pale yellowish-green and showed wilting symptoms. Their relative water content and biomass also decreased [37]. In tomato seedlings, salt stress manifested as yellowing and curling of leaves and a decrease in photosynthetic efficiency, which ultimately led to a reduction in yield and quality [38]. Based on the tissue-specific expression pattern of *SlCCD11* and its potential link to salt stress damage mechanisms, we employed virus-induced gene silencing (VIGS) to silence *SlCCD11* in tomato seedlings to investigate its role in salt tolerance. We found that under salt stress, *SlCCD11*-silenced plants exhibited significant reductions in plant height, stem diameter, fresh weight, and dry weight (Figure 7). This demonstrated that silencing of *SlCCD11* inhibited plant growth under salt stress. As the severity of salt stress increases, both growth parameters and chlorophyll content in tomato seedlings were significantly reduced [39]. For instance, knock-out of *StTST3.1* in potato leaves resulted in decreased chlorophyll content and impaired photosynthesis, thereby inhibiting plant growth [40]. In our study, silencing of *SlCCD11* was also found to reduce chlorophyll content under salt stress (Figure 9). In addition, we discovered that under salt stress conditions, *SlCCD11* can respond to the expression of genes *SlCH1* and *SlCH2* related to chlorophyll synthesis was repressed in *SlCCD11*-silenced leaves. These results suggest that suppression of *SlCCD11* expression increases the sensitivity of tomato seedlings to salt stress. These results suggest that the loss of *SlCCD11* function impairs the tomato’s ability to cope with multi-level physiological disruptions caused by salt stress, thereby increasing seedling sensitivity and susceptibility to salinity. However, the mechanism between them still requires further in-depth study.

Additionally, plant *CCDs* are known to be involved in the degradation of carotenoids. Previous studies have demonstrated that *CCDs* catalyze the oxidative cleavage of carotenoids in plants, leading to their degradation and the production of various apocarotenoids, including ABA, SLs, and volatile compounds [11]. These apocarotenoids play an important role in plant growth and development and respond to environmental stimuli [11]. For instance, suppression of *NtCCD1* in tobacco leaves enhanced carotenoid accumulation [15]. Similarly, inhibition of *CCD4* in chrysanthemum (*Chrysanthemum* × *morifolium*) and Japanese morning glory (*Ipomoea purpurea*) induced carotenoid accumulation, resulting in yellow pigmentation in originally white cultivars [41]. Furthermore, in our study, silencing of *SlCCD11* led to the accumulation of carotenoids in leaves under salt stress. This metabolic change triggered a more severe leaf chlorosis and phenomenon under salt stress (Figure 7A). In addition, our results provide direct functional evidence that suppression of *SlCCD11* in tomatoes reduced the survival rate of salt-treated plants (Figure 8). This phenotype paralleled the well-characterized dwarfism and increased branching seen in *occcd7* and *ntccd8* mutants of rice and tobacco [30,42]. Therefore, we hypothesize that *SlCCD11* can affect the distribution of cytochromes under salt stress conditions by promoting the degradation of carotenoids. In soybean and rapeseed, the expression of specific *CCD* genes (e.g., *GmCCD1*, *GmCCD4*, *BnCCD1*, and *BnCCD4*) could be co-induced by both salt stress and ABA treatment [21,22]. This co-induction pattern suggests that these *CCD* genes may be integrated into the regulatory network linking salt stress and ABA signals, although their precise molecular mechanisms still require direct validation through functional experiments. Specifically, in plants, *NCED* is the key rate-limiting enzyme in ABA biosynthesis, capable of specifically cleaving 9-cis-epoxycarotenoids to generate ABA precursors. Furthermore, *NCED* activity was induced by salt stress, thereby promoting ABA synthesis under stress conditions [43]. Here, we also found that silencing of *SlCCD11* led to reduced expression levels of the key ABA biosynthetic genes *SlNCED1* and *SlNCED2* (Figure 10). Based on the above results, a hypothesis can be drawn that *SlCCD11* may promote ABA synthesis by upregulating the expression of *SlNCEDs*, thereby enhancing the salt tolerance of tomato seedlings. However, the precise mechanism concerning CCD-directed ABA production under saline stress requires further experimental verification.

In plants, SOD catalyzes the dismutation of O_2_·^−^ into H_2_O_2_, which is then decomposed into water by POD [44]. Under salt stress, plants upregulate these enzymes to mitigate oxidative damage by scavenging reactive ROS and maintaining redox homeostasis [45]. For example, in cotton, knockdown of *GhCNGC32* and *GhCNGC35* genes led to a decrease in POD activity and a significant increase in malondialdehyde (MDA) content under salt stress, indicating a crucial role of POD in weakened antioxidant capacity [46]. In this study, the content of H_2_O_2_ and O_2_·^−^ increased (Figure 11A,B) while the activities of SOD and POD enzymes decreased in tomato plants suffering from *SlCCD11* gene silencing (Figure 11C,D). These results suggest that *SlCCD11* may play a positive regulatory role in the tomato salt stress response, assisting in maintaining oxidative homeostasis by influencing the activities of antioxidant enzymes. Usually, ABA can alleviate the damage caused by salt stress by enhancing the antioxidant effect and promoting the clearance of reactive ROS [3]. In chili pepper (*Capsicum annuum* L.), the expression levels of *CaCCD1A* and *CaCCD1B* genes were highest in fruits and increased during ripening, concomitant with the accumulation of β-ionone [47]. Notably, β-ionone and other *CCD* cleavage products have been independently shown in other plant systems to possess free radical scavenging capacity or to induce antioxidant defense systems, which may confer tolerance to abiotic stresses [48]. This study demonstrates that *SlCCD11* expression is upregulated under salt stress and functions through the cleavage of carotenoids. Moreover, the carotenoid metabolic pathway itself possesses inherent antioxidant capacity. Therefore, we speculate that *SlCCD11* may enhance cellular oxidative stress tolerance and improve plant salt tolerance. Therefore, *SlCCD11* may regulate the expression of *NCED* to promote ABA synthesis, thereby enhancing the activity of antioxidant enzymes to enhance salt tolerance. However, the synergistic regulatory mechanism of *SlCCD11* on the activity of antioxidant enzymes still requires further in-depth study. In conclusion, as a positive regulator, *SlCCD11* plays a crucial role under salt stress through its core enzymatic activity of cleaving carotenoids. It likely helps tomato plants coordinate osmotic adjustment and oxidative defense by promoting ABA biosynthesis, thereby maintaining physiological homeostasis and enhancing the survival rate in saline conditions. This highlights the potential of the *SlCCD11* gene and its regulatory pathway in crop stress-resistance improvement; enhancing its function could be of significant importance for molecular breeding programs aimed at developing tomato varieties with stronger salinity tolerance and sustained high Survival rate. However, the mechanism between them still requires further in-depth study.

## 4. Materials and Methods

### 4.1. Genome-Wide Identification of SlCCD Family Members

We downloaded the tomato genome sequence and the ITAG4.0 annotation from Phytozome 13 (https://phytozome.jgi.doe.gov/pz/, accessed on 17 January 2025) [49]. Subsequently, we extracted the protein sequences of tomato CCDs using the ‘GXF sequence extract’ and ‘Batch Translate CDS’ functions in TBtools 4 (Toolbox for ecological battle) v2.034 software. The *A. thaliana* CCD protein sequences were sourced from TAIR (https://www.arabidopsis.org/index.jsp, accessed on 19 January 2025). A BLAST alignment analysis was conducted utilizing the ‘BLAST GUI Wrapper’ module in TBtools, which employed NCBI BLAST+ (version 2.13.0) for the alignments. This was followed by reciprocal BLAST verification against the NCBI database (https://www.ncbi.nlm.nih.gov/, accessed on 20 January 2025). The potential gene family members of tomato *SlCCD* were identified using the ‘Blast XML to Table’ function in TBtools software (version 2.034). Finally, the domains of tomato *SlCCD* proteins were validated with the SMART tool (http://smart.embl-heidelberg.de/, accessed on 23 January 2025), and *SlCCD* members were selected based on conserved domains [50,51].

### 4.2. Conserved Motifs and Chromosomal Localization of SlCCD Family Members

The gene structure of *SlCCD* members was analyzed utilizing the ‘Visualize Gene Structure (from GTF/GFF3 File)’ function in TBtools software (v2.034) [52]. Gene locations were visualized through the ’Gene Location Visualization from GTF/GFF file’ function [53]. Subsequently, each *SlCCD* gene member was assigned to its corresponding position on the tomato genome’s chromosome and renamed accordingly based on the chromosome sequence.

### 4.3. Phylogenetic Tree and Cis-Acting Elements Analysis

The protein sequences of CCD from *A. thaliana*, rice (*Oryza sativa*), and potato (*Solanum tuberosum* L.) were obtained from the TAIR and Phytotome v13 databases (https://phytozome.jgi.doe.gov/pz/, accessed on 12 February 2025). A phylogenetic tree of CCDs was constructed using the Neighbor-Joining method in MEGA 7.0 (version 7.0.26) with a Bootstrap parameter of 1000 [54]. Subsequently, the EvolView (https://evolgenius.info//evolview-v2/#login, accessed on 19 February 2025) online tool was utilized to enhance the visualization of the tree for improved readability and aesthetics.

The transcriptional regulation features of *SlCCD* genes in tomato were systematically examined through bioinformatics approaches. A 2 kb promoter sequence preceding each *SlCCD* gene was extracted and scrutinized. The sequences underwent analysis via the Plant CARE online tool (https://bioinformatics.psb.ugent.be/webtools/plantcare/html/, accessed on 25 February 2025) and were visualized using TBtools software.

### 4.4. Tissue-Specific Expression Analysis

We initially utilized the IDs of the *SlCCD* gene family members to extract tissue-specific expression data from the eFP database (http://bar.utoronto.ca/efp/cgi-bin/efpWeb.cgi, accessed on 12 March 2025). Subsequently, we compiled the expression data for the SlCCDs across various tomato tissues and visualized the tissue-specific expression patterns of the *SlCCD* gene family members using TBtools. Furthermore, we examined the gene expression of the *SlCCD* family members in the root, stem, and leaf tissues of tomato.

### 4.5. Plant Material and Growing Conditions

The tomato variety chosen for this study is ‘Micro-Tom’, known for its compact size, short growth cycle, small fruits, and high degree of self-fertilization. Initially, ‘Micro-Tom’ tomato seeds were carefully selected and placed in a conical flask with approximately 100 mL of sterile water. The flask was then placed on an HYG-C shaker, rotating at 180 r min^−1^, and incubated at 25 °C for 2–3 d, with daily water changes. Upon germination, the sprouted seeds were transferred to a tray filled with a mixture of peat and vermiculite nutrient soil in a 2:3 ratio and moved to a plant growth chamber. The growth chamber maintained a light intensity of 250 m^−2^ s^−1^, with a 16 h day temperature of 26 ± 2 °C and an 8 h night temperature of 20 ± 2 °C, while sustaining a relative humidity of 60%. Following a two-week cultivation period in the growth chamber, seedlings were relocated from the nutrient soil to a hydroponic nutrient solution for further growth. After 21 d, seedlings of uniform size were selected for subsequent experimentation [55].

### 4.6. Stress Treatment and Tissue Expression

Seedlings were subjected to treatments with abscisic acid (ABA), MeJA, sodium chloride (NaCl), and light. Selected seedlings were subsequently transferred to a Hogland nutrient solution containing 150 mM NaCl, 100 μM ABA, and 100 μM MeJA, while maintaining identical conditions to those in the growth chamber. After 6, 12, and 24 h, the harvested leaves were rapidly frozen in liquid nitrogen and stored at −80 °C for future analysis. The plants at 0 h served as the control (CK) group. The preservation methods for materials in both the control and experimental groups were consistent. Each treatment included three biological replicates, with eight strains per replicate. All plants were assessed at the same seedling stage to ensure that the treatment effects were evaluated under uniform physiological conditions [56]. In addition, the roots, stems, and leaves of the untreated 21-day-old seedlings were collected for the analysis of tissue expression. Each treatment group consisted of 3 biological replicates, and each replicate group contained 8 plants.

### 4.7. RNA Extraction and qRT-PCR

Total RNA was isolated from samples using TRIzol reagent (Invitrogen, Carlsbad, CA, USA). The extraction procedure involved grinding 0.5 g of leaves into powder with liquid nitrogen, transferring the powder to 1.5 mL centrifuge tubes, adding 1 mL of TRIzol, incubating on ice for 5 min, centrifuging at 12,000 rpm for 20 min at 4 °C, and recovering the supernatant. Subsequently, an equal volume of isopropyl alcohol was added, gently mixed, and incubated at −20 °C for 1 h. The solution was then transferred to an adsorption column, centrifuged at 12,000 rpm for 15 min at 4 °C, washed twice with 700 μL of 75% ethanol (1 min per centrifugation), and finally centrifuged in air for 2 min. After drying the pellet for 40 min, 35 μL of RNase-free water was added, incubated for 2 min, centrifuged, eluted twice, and the RNA was collected [56]. Subsequently, the FastQuant First Strand cDNA Synthesis Kit (Tianen, Beijing, China) was utilized for cDNA synthesis. For quantitative real-time PCR (qRT-PCR), the SYBR Green Premix Pro Taq HSPremix kit was employed with a LightCycler 480 Real-Time PCR System (Roche Applied Science, Penzberg, Germany) [57]. The primer sequences can be found in Table S1. For detailed methodologies, please refer to [58].

### 4.8. VIGS Vector Construction and Its Genetic Transformation

To examine the role of the *SlCCD11* gene, we generated a vector using the TRV-mediated virus-induced gene silencing (VIGS) method. Utilizing tomato cDNA as a template and specific primers targeting Xba 1 and Kpn 1 restriction sites (Table S2), we amplified a 300 bp fragment of the *SlCCD11* gene and inserted it into the TRV2 vector to create the pTRV2-*SlCCD11* recombinant plasmid (Figure S1A). The accuracy of the vector construction was confirmed through enzyme digestion and sequencing validation (Figure S1B). Subsequently, the empty pTRV2, helper vector pTRV1, and the recombinant plasmid pTRV2-*SlCCD11* were separately introduced into competent *Agrobacterium tumefaciens* GV3101 cells. In the infection trial, two treatment groups were established: (1) a negative control group in which hypocotyls of tomato seedlings at the early seedling stage (two cotyledons fully expanded and the first true leaf just visible) were inoculated with a mixture of pTRV1 and empty pTRV2 at a 1:1 volume ratio (OD_600_ = 0.8–1.1); and (2) a gene silencing group in which cotyledons of the seedlings at this identical developmental stage were infiltrated with a mixture of pTRV1 and pTRV2-*SlCCD11* at the same ratio. The phenotypic changes were assessed seven days post-infection, and the first and second true leaves were harvested for gene silencing efficiency evaluation via qRT-PCR. Ultimately, quantitative analysis revealed a 79.8% reduction in the silencing efficiency of the *SlCCD11* plants (Figure S2), confirming the successful gene silencing and enabling subsequent investigations.

### 4.9. NaCl Treatment and Survival Rate of SlCCD11-Silenced Plants

Once the seedlings reached 21 d old (at the three-leaf-one-heart stage), they were treated with a nutrient solution containing 150 mM NaCl, and the plants treated with only the nutrient solution were used as CK. After 7 d of NaCl treatment, seedlings were photographed, and a subset was collected and stored for subsequent measurement of relevant physiological indicators. The remaining seedlings continued to be treated with the 150 mM NaCl nutrient solution for an additional 7 d. At the end of this period, photographs were taken again, and the seedling survival rate was calculated.

### 4.10. Growth Phenotypes

Measure the plant height by using a vernier caliper to determine the distance from the base to the top growth point. Define this linear distance as the plant height. Utilize a vernier caliper to measure the diameter of the plant stem. Cleanse the seedling roots to eliminate impurities and remove excess water. Weigh the seedlings to determine their fresh weight. Subsequently, dry the plants in an 80 °C oven for 48 h. Measure the weight of the dried plants to ascertain the dry weight [59].

### 4.11. Measurement of Chlorophyll and Carotenoid Contents

Weigh 0.1 g of tomato leaves and cut them up (taking care to avoid the main leaf veins). Place the leaf in a container containing 10 mL of 80% acetone solution and make sure the leaf is completely submerged. Then, the container was placed in a dark environment for extraction for 48 h until the leaf color completely turned white. After extraction, the extract was thoroughly mixed. Using 80% acetone solution as a blank reference, the absorbance of the extract was measured at 663 nm, 645 nm, and 440 nm using a spectrophotometer (UV-1800, Shimadzu, Kyoto, Japan) [46].

### 4.12. ROS Content

To determine the hydrogen peroxide (H_2_O_2_) content in tomato leaves, 0.5 g of the leaves is mixed with acetone in a pre-cooled mortar, homogenized, and adjusted to a volume of 5 mL with pre-cooled acetone. The mixture is then centrifuged for 20 min at 4 °C and 12,000 rpm. Next, 1 mL of the supernatant is combined with 0.1 mL of TiCl_4_ and 0.2 mL of concentrated ammonia water in a new centrifuge tube, thoroughly mixed, and centrifuged at 5000 rpm for 10 min. The supernatant is discarded, and the pigment is eliminated. The resulting precipitate is washed with acetone 2–3 times. Subsequently, 3 mL of 2 mol·L^−1^ H_2_SO_4_ is added to dissolve the precipitate, and after complete dissolution, the absorbance is measured at 415 nm.

Superoxide anions (O_2_·^−^) Content. Leaves weighing 0.5 g were ground in PBS (0.05 mol·L^−1^, pH 7.8) in an ice bath, diluted to 5 mL, and then centrifuged at 4 °C, 12,000 rpm for 20 min to obtain the crude enzyme extract in the supernatant. To a centrifuge tube, 0.5 mL of the extract, 0.5 mL of phosphate buffer (PBS), and 1 mL of hydroxylamine hydrochloride (1 mmol·L^−1^) were added, mixed thoroughly, and incubated at 25 °C for 1 h. Subsequently, 1 mL of 1-naphthylamine (7 mmol·L^−1^) and 1 mL of sulfanilic acid (17 mmol·L^−1^) were added, vortexed, incubated at 25 °C for 20 min, and the absorbance at 530 nm was measured [60].

### 4.13. Antioxidant Enzyme Activities

Enzyme extraction: Take 0.5 g of tomato leaves and put them in a pre-cooled mortar. Add 0.1 g of PVP, a small amount of quartz sand, and 1 mL of pre-cooled 50 mM PBS (pH 7.8). Grind them into a homogeneous mixture and then dilute to 5 mL. Centrifuge at 4 °C and 10,000 rpm for 15 min, then take the supernatant. Store it at 4 °C for future use. Superoxide dismutase (SOD) activity: In test tubes, sequentially add 1.5 mL of 0.05 mol/L phosphate buffer (pH 7.8), 0.3 mL of 130 mmol/L methionine solution 0.3 mL of 750 μmol/L nitroblue tetrazolium (NBT) solution, 0.3 mL of 100 μmol/L EDTA-Na_2_ solution, 0.3 mL of 20 μmol/L riboflavin solution, 0.2 mL of enzyme extract, and 0.28 mL of distilled water PBS was added to the blank tubes and the control tubes instead of the enzyme solution. After mixing the reagents, the blank tubes were immediately placed in the dark, while the other test tubes were exposed to 4000 lx light for 20 min for the reaction. After the reaction period, measure absorbance at 560 nm using the blank as a reference. Peroxidase (POD) determination: Zero the instrument with distilled water. First, add 0.1 mL of the test solution (the control tube uses the extraction buffer instead of the enzyme solution) to the cuvette. Then, add 2.6 mL of 0.3% guaiacol. After placing the cuvette in the colorimeter, add 0.3 mL of 0.6% H_2_O_2_. Immediately record the change in absorbance at 470 nm within 2 min [60].

## 5. Conclusions

In conclusion, a total of 12 members were identified in the tomato *SlCCD* family. The research has found that the *SlCCD* genes in tomato are highly conserved and play a significant role in stress responses and hormone responses. The analysis of tissue-specific expression showed that the *SlCCD11* gene was highly expressed in the leaves of tomatoes. Furthermore, the promoter region of the *SlCCD* genes contains multiple *cis*-acting elements that respond to hormones and stresses. *SlCCD11* exhibited the most significant response under salt treatment. Further functional analysis indicates that *SlCCD11* is a positive regulator of salt stress response. Under salt stress, the transient silencing of *SlCCD11* led to an increase in total carotenoid and reactive oxygen species content, while the chlorophyll content, antioxidant enzyme activity, and expression levels of ABA-related genes decreased. Therefore, *SlCCD11* may promote the synthesis of ABA by facilitating the degradation of carotenoids or by upregulating the expression of *SlNCEDs*, thereby enhancing the salt tolerance of tomato seedlings. However, the precise mechanism of *SlCCDs* through ABA in salt stress remains to be uncovered. Furthermore, in future work, the function of *SlCCD11* should be verified by overexpressing it in plants and analyzing its effects on ABA synthesis and carotenoid metabolism. Additionally, using techniques such as yeast two-hybrid screening to identify its interacting proteins will help clarify the molecular regulatory mechanism of *SlCCD11*. Exploring its synergistic effects with key salt-tolerance pathways (e.g., SOS and NHX) to systematically determine the node function of *SlCCD11* in the tomato salt-tolerance network will provide new candidate genes and a theoretical basis for breeding salt-tolerant tomatoes.

## Figures and Tables

**Table 1:** Physical and chemical properties of the tomato *SlCCD* gene family.

Gene	Gene ID	Gene Locus	Amino Acid	Molecular Weight (Da)	PI	GRAVY
*SlCCD1*	*Solyc01g087250.3.1.ITAG4.0*	Chr01	545	61,201.22	6.14	−0.272
*SlCCD2*	*Solyc01g087260.4.1.ITAG4.0*	Chr01	562	63,252.40	5.98	−0.372
*SlCCD3*	*Solyc01g087270.3.1.ITAG4.0*	Chr01	86	9548.05	6.57	−0.534
*SlCCD4*	*Solyc01g090660.3.1.ITAG4.0*	Chr01	678	76,853.95	8.25	−0.368
*SlCCD5*	*Solyc05g053530.1.1.ITAG4.0*	Chr05	583	64,960.30	8.21	−0.341
*SlCCD6*	*Solyc07g056570.1.1.ITAG4.0*	Chr07	605	67,317.33	6.25	−0.406
*SlCCD7*	*Solyc08g016720.1.1.ITAG4.0*	Chr08	581	64,833.54	6.07	−0.333
*SlCCD8*	*Solyc08g066650.3.1.ITAG4.0*	Chr08	557	62,073.99	6.37	−0.313
*SlCCD9*	*Solyc08g066720.3.1.ITAG4.0*	Chr08	414	47,598.51	5.99	−0.300
*SlCCD10*	*Solyc08g075480.4.1.ITAG4.0*	Chr08	577	64,083.37	8.23	−0.155
*SlCCD11*	*Solyc08g075490.4.1.ITAG4.0*	Chr08	616	68,030.64	7.31	−0.224
*SlCCD12*	*Solyc11g071227.1.1.ITAG4.0*	Chr11	598	68,857.20	6.93	−0.261

Gene IDs were retrieved from the NCBI database, and the corresponding physicochemical properties of these genes were recalculated using the TB tool.

## Data Availability

Data are contained within the article or Supplementary Materials.

## Supplementary Materials

The following supporting information can be downloaded at: https://www.mdpi.com/article/10.3390/plants15020300/s1, Figure S1. TRV:SlCCD11 construction of a silent vector. (**A**) Amplification of the *SlCCD11* gene; (**B**) PCR detection of bacterial solution; M: DNA Marker, 1–2: PCR amplification product. 1–4: PCR detection of bacterial solution. Figure S2. Silencing efficiency of *SlCCD11* detected by qPCR. Figure S3. Expression analysis of *SlCCD* in different tissues of tomatoes. Different numbers represent significance levels, and the map indicates low/medium/high expression in blue/yellow/red, respectively. Table S1 Primer sequences of *CCD*, chlorophyll synthesis, and ABA-related genes. Table S2. TRV:SlCCD11-specific primer fragment sequence.

## References

[1] Apostolova E.L. (2023). Molecular Mechanisms of Plant Defense against Abiotic Stress. Int. J. Mol. Sci..

[2] Lv R., Mo F., Li C., Meng F., Zhang H., Yu L., Cheng M., Wang P., Liu S., Liu Z. (2024). Genome-wide identification of the *CLC* gene family in tomato (*Solanum lycopersicum*) and functional analysis of *SlCLC8* in salt stress tolerance. Sci. Hortic..

[3] Li S., Liu S., Zhang Q., Cui M., Zhao M., Li N., Wang S., Wu R., Zhang L., Cao Y. (2022). The interaction of ABA and ROS in plant growth and stress resistances. Front. Plant Sci..

[4] Yao Y., Jia L., Cheng Y., Ruan M., Ye Q., Wang R., Yao Z., Zhou G., Liu J., Yu J. (2022). Evolutionary Origin of the Carotenoid Cleavage Oxygenase Family in Plants and Expression of Pepper Genes in Response to Abiotic Stresses. Front. Plant Sci..

[5] Ahrazem O., Gómez-Gómez L., Rodrigo M.J., Avalos J., Limón M.C. (2016). Carotenoid Cleavage Oxygenases from Microbes and Photosynthetic Organisms: Features and Functions. Int. J. Mol. Sci..

[6] Magome H., Arai M., Oyama K., Nishiguchi R., Takakura Y. (2023). Multiple loss-of-function mutations of carotenoid cleavage dioxygenase 4 reveal its major role in both carotenoid level and apocarotenoid composition in flue-cured mature tobacco leaves. Sci. Rep..

[7] Dhar M.K., Mishra S., Bhat A., Chib S., Kaul S. (2020). Plant carotenoid cleavage oxygenases: Structure-function relationships and role in development and metabolism. Brief. Funct. Genom..

[8] Liu L., Shao Z., Zhang M., Wang Q. (2015). Regulation of carotenoid metabolism in tomato. Mol. Plant.

[9] Walter M.H., Floss D.S., Strack D. (2010). Apocarotenoids: Hormones, mycorrhizal metabolites and aroma volatiles. Planta.

[10] Wang J.Y., Haider I., Jamil M., Fiorilli V., Saito Y., Mi J., Baz L., Kountche B.A., Jia K.P., Guo X. (2019). The apocarotenoid metabolite zaxinone regulates growth and strigolactone biosynthesis in rice. Nat. Commun..

[11] Qi Z., Fan X., Zhu C., Chang D., Pei J., Zhao L. (2022). Overexpression and Characterization of a Novel Plant Carotenoid Cleavage Dioxygenase 1 from Morus notabilis. Chem. Biodivers..

[12] Choi H., Yi T.G., Gho Y.S., Kim J.H., Kim S., Choi Y.J., Lim S., Eom S.H., Jung K.H., Ha S.H. (2025). Augmenting carotenoid accumulation by multiplex genome editing of the redundant CCD family in rice. Plant Physiol. Biochem..

[13] Wei Y., Wan H., Wu Z., Wang R., Ruan M., Ye Q., Li Z., Zhou G., Yao Z., Yang Y. (2016). A comprehensive analysis of carotenoid cleavage dioxygenases genes in *Solanum lycopersicum*. Plant Mol. Biol. Rep..

[14] Zhong Y., Pan X., Wang R., Xu J., Guo J., Yang T., Zhao J., Nadeem F., Liu X., Shan H. (2020). *ZmCCD10a* Encodes a Distinct Type of Carotenoid Cleavage Dioxygenase and Enhances Plant Tolerance to Low Phosphate. Plant Physiol..

[15] Du F., Hu Z., Qin L., Zhang C., Wang Z., Shi Y., Wang X., Wang R., Gao Y., Dong C. (2023). Suppression of carotenoid cleavage dioxygenase 1 (*NtCCD1*) increases carotenoid contents and attenuates reactive oxygen species (ROS) in Tobacco Leaves. Plant Growth Regul..

[16] Frusciante S., Diretto G., Bruno M., Ferrante P., Pietrella M., Prado-Cabrero A., Rubio-Moraga A., Beyer P., Gomez-Gomez L., Al-Babili S. (2014). Novel carotenoid cleavage dioxygenase catalyzes the first dedicated step in saffron crocin biosynthesis. Proc. Natl. Acad. Sci. USA.

[17] Wang Y., Xu J., Liu A. (2022). Identification of the carotenoid cleavage dioxygenase genes and functional analysis reveal *DoCCD1* is potentially involved in beta-ionone formation in *Dendrobium officinale*. Front. Plant Sci..

[18] Wang J., Zhang N., Zhao M., Jing T., Jin J., Wu B., Wan X., Schwab W., Song C. (2020). Carotenoid Cleavage Dioxygenase 4 Catalyzes the Formation of Carotenoid-Derived Volatile β-Ionone during Tea (*Camellia sinensis*) Withering. JAF Chem..

[19] Sarwar S., Sami A., Haider M.Z., Tasawar L., Akram J., Ahmad A., Shafiq M., Zaki H.E.M., Ondrasek G., Shahid M.S. (2025). Genome-Wide Identification and In Silico Expression Analysis of *CCO* Gene Family in *Citrus clementina* (Citrus) in Response to Abiotic Stress. Plants.

[20] Mishra S., Upadhyay S., Shukla R.K. (2017). The Role of Strigolactones and Their Potential Cross-talk under Hostile Ecological Conditions in Plants. Front. Physiol..

[21] Wang R., Wang C., Fei Y., Gai J., Zhao T. (2013). Genome-wide identification and transcription analysis of soybean carotenoid oxygenase genes during abiotic stress treatments. Mol. Biol. Rep..

[22] Zhou X.T., Jia L.D., Duan M.Z., Chen X., Qiao C.L., Ma J.Q., Zhang C., Jing F.Y., Zhang S.S., Yang B. (2020). Genome-wide identification and expression profiling of the carotenoid cleavage dioxygenase (*CCD*) gene family in *Brassica napus* L.. PLoS ONE.

[23] Zhao J., Li J., Zhang J., Chen D., Zhang H., Liu C., Qin G. (2021). Genome-wide identification and expression analysis of the carotenoid cleavage oxygenase gene family in five rosaceae species. Plant Mol. Biol. Rep..

[24] Tantisuwanichkul K., Sirikantaramas S. (2024). Genome-wide analysis of carotenoid cleavage oxygenases and identification of ripening-associated *DzNCED5a* in durian (*Durio zibethinus*) fruit. Plant Physiol. Biochem..

[25] Yue X.Q., Zhang Y., Yang C., Li J., Rui X., Ding F., Hu F., Wang X., Ma W., Zhou K. (2022). Genome-wide identification and expression analysis of carotenoid cleavage oxygenase genes in Litchi (*Litchi chinensis* Sonn.). BMC Plant Biol..

[26] Ding A., Bao F., Cheng W., Cheng T., Zhang Q. (2023). Phylogeny of *PmCCD* Gene Family and Expression Analysis of Flower Coloration and Stress Response in *Prunus mume*. Int. J. Mol. Sci..

[27] Yao Z., Duan W., Li A., Zhan W., Sun S., Pan L., Niu L., Cui G., Zeng W. (2025). Genome-wide identification of *CCD* gene family in Peach (*Prunus persica* L. Batsch) and expression analysis with aroma norisoprenoids. BMC Plant Biol..

[28] Zhou Q., Li Q., Li P., Zhang S., Liu C., Jin J., Cao P., Yang Y. (2019). Carotenoid Cleavage Dioxygenases: Identification, Expression, and Evolutionary Analysis of This Gene Family in Tobacco. Int. J. Mol. Sci..

[29] Cheng D., Wang Z., Li S., Zhao J., Wei C., Zhang Y. (2022). Genome-Wide Identification of *CCD* Gene Family in Six *Cucurbitaceae* Species and Its Expression Profiles in Melon. Genes.

[30] Gao J., Zhang T., Xu B., Jia L., Xiao B., Liu H., Liu L., Yan H., Xia Q. (2018). CRISPR/Cas9-mediated mutagenesis of carotenoid cleavage dioxygenase 8 (*CCD8*) in tobacco affects shoot and root architecture. Int. J. Mol. Sci..

[31] Zhang S., Guo Y., Zhang Y., Guo J., Li K., Fu W., Jia Z., Li W., Tran L.P., Jia K.P. (2021). Genome-wide identification, characterization and expression profiles of the *CCD* gene family in *Gossypium* species. 3 Biotech.

[32] Kapli P., Yang Z., Telford M.J. (2020). Phylogenetic tree building in the genomic age. Nat. Rev. Genet..

[33] Panchy N., Lehti-Shiu M., Shiu S.H. (2016). Evolution of Gene Duplication in Plants. Plant Physiol..

[34] Yamaguchi-Shinozaki K., Shinozaki K. (2005). Organization of Cis-acting regulatory elements in osmotic- and cold-stress-responsive promoters. Trends Plant Sci..

[35] Li L., Huang Z., Zhang Y., Mu Y., Li Y., Nie L. (2025). Regulation of 2-acetyl-1-pyrroline (2-AP) biosynthesis and grain quality in fragrant rice under salt stress. Field Crops Res..

[36] Hmissi M., Chaieb M., Krouma A. (2025). Exogenous IAA application enhances durum wheat tolerance to salinity by regulating osmotic adjustment and ionic homeosta sis. Russ. J. Plant Physiol..

[37] Ma C., Bian C., Liu W., Sun Z., Xi X., Guo D., Liu X., Tian Y., Wang C., Zheng X. (2022). Strigolactone alleviates the salinity-alkalinity stress of Malus hupehensis seedlings. Front. Plant Sci..

[38] Raziq A., Mohi Ud Din A., Anwar S., Wang Y., Jahan M.S., He M., Ling C., Sun J., Shu S., Guo S. (2022). Exogenous spermidine modulates polyamine metabolism and improves stress responsive mechanisms to protect tomato seedlings against salt stress. Plant Physiol. Biochem..

[39] Liu T., Kawochar M.A., Liu S., Cheng Y., Begum S., Wang E., Zhou T., Liu T., Cai X., Song B. (2023). Suppression of the tonoplast sugar transporter, *StTST3.1*, affects transitory starch turnover and plant growth in potato. Plant J..

[40] Ohmiya A., Kishimoto S., Aida R., Yoshioka S., Sumitomo K. (2006). Carotenoid cleavage dioxygenase (*CmCCD4a*) contributes to white color formation in chrysanthemum petals. Plant Physiol..

[41] Kulkarni K.P., Vishwakarma C., Sahoo S.P., Lima J.M., Nath M., Dokku P., Gacche R.N., Mohapatra T., Robin S., Sarla N. (2014). A substitution mutation in *OsCCD7* cosegregates with dwarf and increased tillering phenotype in rice. J. Genet..

[42] Basso M.F., Contaldi F., Celso F.L., Karalija E., Paz-Carrasco L.C., Barone G., Ferrante A., Martinelli F. (2023). Expression profile of the *NCED*/*CCD* genes in chickpea and lentil during abiotic stress reveals a positive correlation with increased plant tolerance. Plant Sci..

[43] Zhou Y., Li S., Ran S., Xu Y., Hou M., Han M., Zhong F. (2024). Genome-Wide Identification and Characterization of the Superoxide Dismutase (SOD) Gene Family in Pakchoi and the Role of the BchFSD2 Gene in the Salt Stress Toleran. Agronomy.

[44] Hasanuzzaman M., Raihan M.R.H., Nowroz F., Fujita M. (2022). Insight into the Mechanism of Salt-Induced Oxidative Stress Tolerance in Soybean by the Application of Bacillus subtilis: Coordinated Actions of Osmoregulation, Ion Homeostasis, Antioxidant Defense, and Methylglyoxal Detoxification. Antioxidants.

[45] Lu Z., Yin G., Chai M., Sun L., Wei H., Chen J., Yang Y., Fu X., Li S. (2022). Systematic analysis of CNGCs in cotton and the positive role of *GhCNGC32* and *GhCNGC35* in salt tolerance. BMC Genom..

[46] Zhang Z., Hou X., Gao R., Li Y., Ding Z., Huang Y., Yao K., Yao Y., Liang C., Liao W. (2024). *CsSHMT3* gene enhances the growth and development in cucumber seedlings under salt stress. Plant Mol. Biol..

[47] Cheng L., Huang N., Jiang S., Li K., Zhuang Z., Wang Q., Lu S. (2021). Cloning and functional characterization of two carotenoid cleavage dioxygenases for ionone biosynthesis in chili pepper (*Capsicum annuum* L.) fruits. Sci. Hortic..

[48] Dos Santos Costa M., de Meireles A.L., Gusevskaya E.V. (2017). Aerobic Palladium-Catalyzed Oxidations in the Upgrading of Biorenewables: Oxidation of β-Ionone and α-Ionone. Asian J. Org. Chem..

[49] Goodstein D.M., Shu S., Howson R., Neupane R., Hayes R.D., Fazo J., Mitros T., Dirks W., Hellsten U., Putnam N. (2012). Phytozome: A comparative platform for green plant genomics. Nucleic Acids Res..

[50] Li Z., Shen J., Liang J. (2019). Genome-Wide Identification, Expression Profile, and Alternative Splicing Analysis of the Brassinosteroid-Signaling Kinase (BSK) Family Genes in *Arabidopsis*. Int. J. Mol. Sci..

[51] Yao K., Yao Y., Ding Z., Pan X., Zheng Y., Huang Y., Zhang Z., Li A., Wang C., Li C. (2023). Characterization of the FLA Gene Family in Tomato (*Solanum lycopersicum* L.) and the Expression Analysis of *SlFLAs* in Response to Hormone and Abiotic Stresses. Int. J. Mol. Sci..

[52] Chen C., Chen H., Zhang Y., Thomas H.R., Frank M.H., He Y., Xia R. (2020). TBtools: An Integrative Toolkit Developed for Interactive Analyses of Big Biological Data. Mol. Plant..

[53] Chen J., Huang X., Salt D.E., Zhao F. (2020). Mutation in *OsCADT1* enhances cadmium tolerance and enriches selenium in rice grain. New Phytol..

[54] Kumar S., Stecher G., Tamura K. (2016). MEGA7: Molecular evolutionary genetics analysis version 7.0 for bigger datasets. Mol. Biol. Evol..

[55] Martí E., Gisbert C., Bishop G.J., Dixon M.S., García-Martínez J.L. (2006). Genetic and physiological characterization of tomato cv. Micro-Tom. J. Exp. Bot..

[56] Wang Y., Liu Z., Li L., Pan X., Yao K., Wei W., Liao W., Wang C. (2024). The Characteristics and Expression Analysis of the Tomato *SlRBOH* Gene Family under Exogenous Phytohormone Treatments and Abiotic Stresses. Int. J. Mol. Sci..

[57] Li L., Liu Z., Pan X., Yao K., Wang Y., Yang T., Huang G., Liao W., Wang C. (2024). Genome-Wide Identification and Characterization of Tomato Fatty Acid β-Oxidase Family Genes *KAT* and *MFP*. Int. J. Mol. Sci..

[58] Schmittgen T.D., Livak K.J. (2008). Analyzing real-time PCR data by the comparative CT method. Nat. Protoc..

[59] Wei L., Zhang J., Wei S., Hu D., Liu Y., Feng L., Li C., Qi N., Wang C., Liao W. (2022). Nitric Oxide Enhanced Salt Stress Tolerance in Tomato Seedlings, Involving Phytohormone Equilibrium and Photosynthesis. Int. J. Mol. Sci..

[60] Liu Y., Wei L., Feng L., Zhang M., Hu D., Tie J., Liao W. (2022). Hydrogen Sulfide Promotes Adventitious Root Development in Cucumber under Salt Stress by Enhancing Antioxidant Ability. Plants.

