# Supplementary material for: Genome-Wide Analysis of Tomato SlCCD Genes and the Role of SlCCD11 in Enhancing Salt Tolerance
Source: Plants (Basel). 2026 Jan 19;15(2):300. doi: 10.3390/plants15020300 (PMC12845110; doi:10.3390/plants15020300)
Supplement: Supplementary file 1 [file plants-15-00300-s001.zip › plants-4033335-supplementary.pdf]

Genome-wide Analysi of Tomato *SlCCD* Genes and the role of *SlCCD11* in Enhancing Salt Tolerance

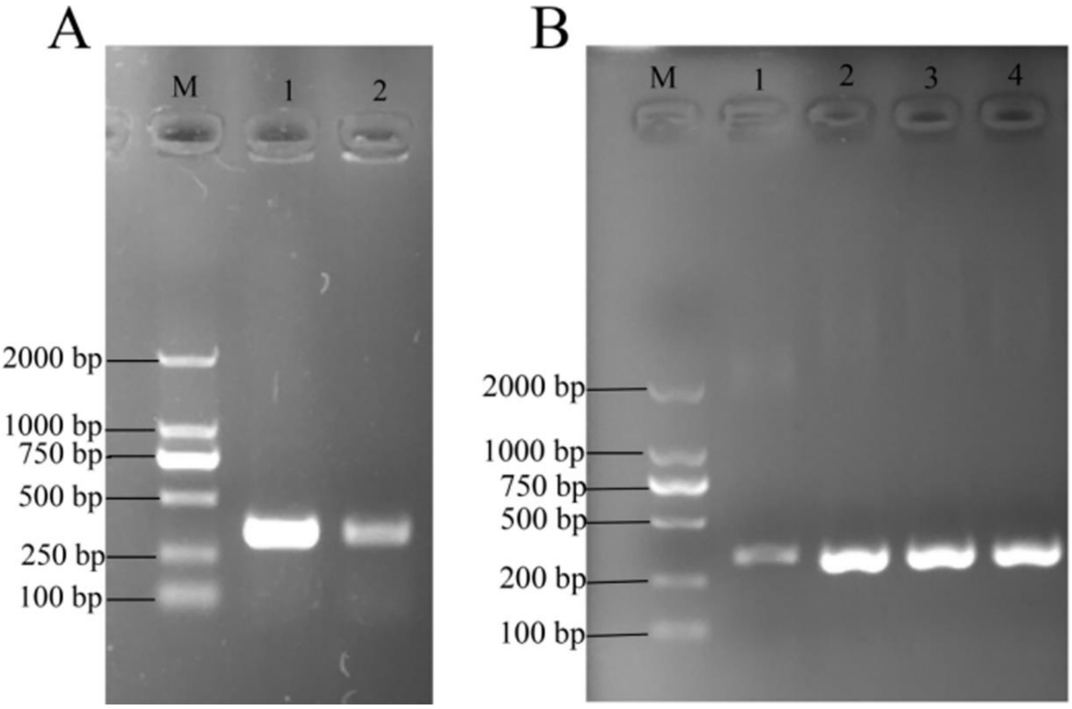

Figure S1. TRV: *SlCCD11* Construction of silent vector. (A) Amplification of the *SlCCD11* gene; (B) PCR detection of bacterial solution; M: DNA Marker, 1-2: PCR amplification product. 1-4: PCR detection of bacterial solution.

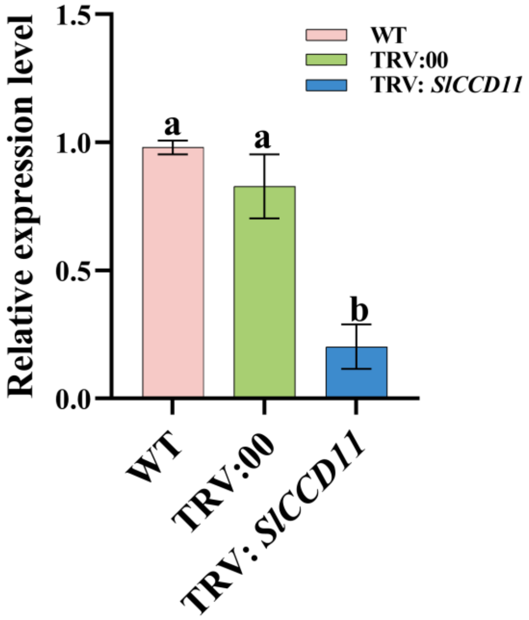

Figure S2. Silencing efficiency of *SlCCD11* detected by qPCR.

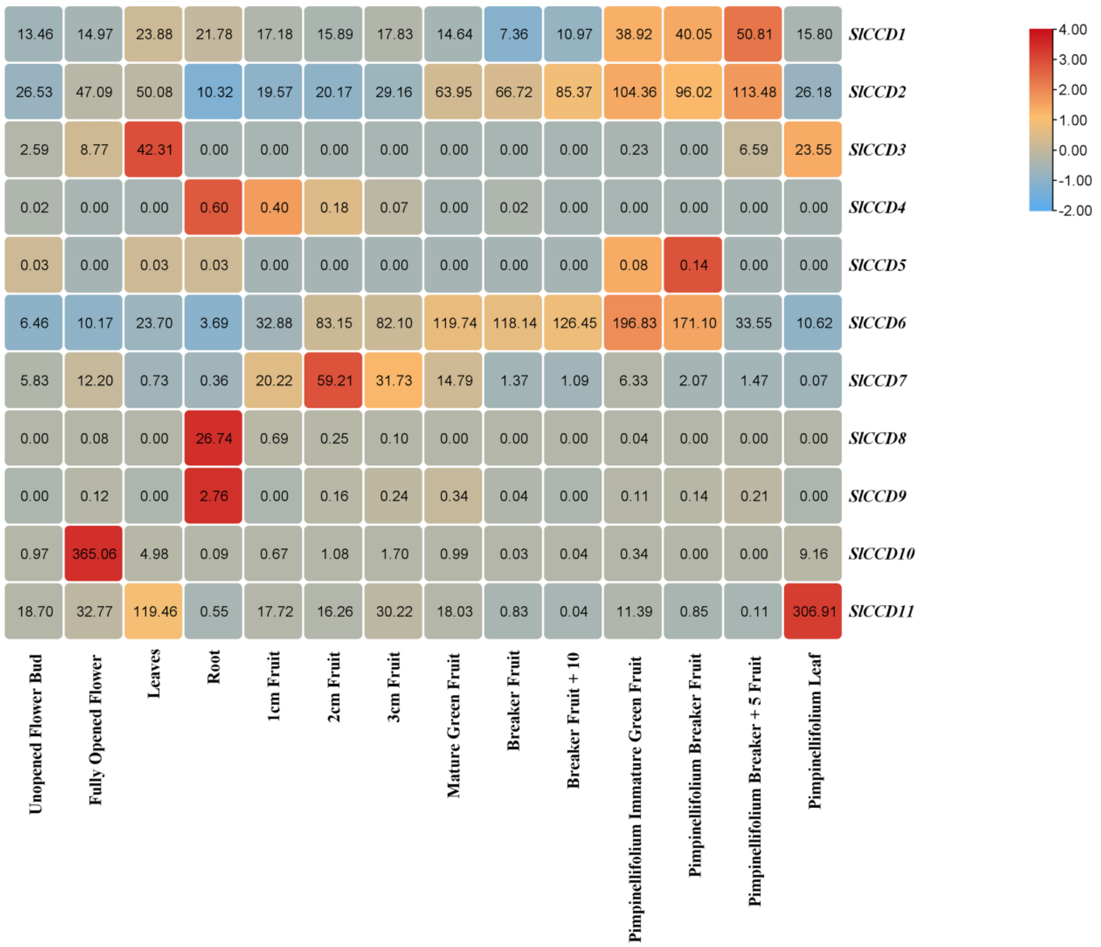

Figure S3. Expression analysis of *SlCCD* in different tissues of tomatoes. Different numbers represent significance levels, and the map indicates low/medium/high expression in blue/yellow/red respectively.

Table S1 Primer sequences of CCD, chlorophyll synthesis and ABA-related genes

Gene	Foward primer (5' - 3')	Reverse primer (5' - 3')
SlActin	TCGTGCTGGATTCTGGTG	GGCAGTGGTGGTGAACAT
SlCCD1	TCCTTCCACGATATGCCAACAATG	AACACGACTTCATCTCCCTCCTC
SlCCD2	ACGCTACGCAAAGAACGAATCC	CGTCATCTCCCTCCTCCCAAG
SlCCD3	GGGGTTGGGTTGTGGTGAAAC	TGGGAATTAGGGACATTGCTTTTG
SlCCD4	TCCATACCATCCGATTTCCCTTCC	CCTTAAGTAGCCGTGTCCGTCTAG
SlCCD5	TCCTCTGTTCAGCACGGTCTTG	AATCGGTTTGTATAACGGGTTGGC
SlCCD6	CCTCTGGAATGCTTGGGAAGAAG	AAATGGAGTCTGGTGGTGTCATAC
SlCCD7	TGCGAGGCTATTGTTGTTCTATGC	AACCAATCCAGCATTAGCAACTCC
SlCCD8	TAGTTCCACCACCAGATCAAGAGG	CTTCAACGACGAGTTCTCCTTCC
SlCCD9	ATGAGGATGATGGTTGGCTTGTG	GATTGTGGTAATAGGGTGTGTTGC
SlCCD10	TATGCCAAGGGTGAATCGGAAATG	ATCGTATCGCCTCCATCCTCTTC
SlCCD11	CGCCCTCGGTGAATCTGATTTAC	GGATGTGCCGTCATGCTCATAG
SlCCD12	GCCACGTTATGGTGATGCTAATTC	CCTCACCACCACCTCATCATAATC

SlCLH1	TCTCGGCATTGATCCTGTTGATGG	CGGAGCACAAGCAGGAAATAGAGG
SlCLH2	TCCGCCATCAGTTCTCACCTACG	CTCCCAAACCCGAGCCAATTACC
SlNCED1	CGTGGGCTCTTCGGACTTGTTG	TTTAAGATCGCCGGTGGGTGTTAC
SlNCED2	AGCAATCAGTCCGGCATAACCTTC	TCGCTCCATTCCTGACATAAACCC

Table S2 TRV:*SlCCD11* specific primer fragment sequence

Enzymatic cleavage site	Primer name	Primers sequence (5'-3')
Xba 1	TRV-*SlCCD11*-F	aaggttaccgaattctctagaACTATTCTCCTACTCTAAAAGTATTTTCTGTTAG
Kpn 1	TRV-*SlCCD11*-R	gagacgcgtgagctcggtaccCTTCGCATTCGGTAGGAGGA
